# Electrical impedance tomography in neonates: a review

**DOI:** 10.1038/s41390-025-03929-x

**Published:** 2025-02-22

**Authors:** Ako A. Ako, Ahmed Ismaiel, Shantanu Rastogi

**Affiliations:** Division of Neonatology, Children’s Hospital at Montefiore, Albert Einstein College of Medicine, Bronx, New York 10467 USA

## Abstract

**Abstract:**

Appropriate interventions informed by real-time assessment of pulmonary function in mechanically ventilated critically ill neonates can reduce the incidence of bronchopulmonary dysplasia, pneumothorax, intraventricular hemorrhage and other complications of newborn life. The respiratory system in neonates is uniquely different from older children, and its physiological and anatomic attributes increase neonatal vulnerability to respiratory distress and eventual failure. While significant advancements have been made in developing respiratory support for neonates, such support is accompanied by inherent risks to their delicate lungs. Ventilator-associated lung injury poses a critical concern that can be potentially decreased with more precise, non-invasive, non-radiating, bedside methods for assessing neonatal pulmonary function in real time. Electrical impedance tomography (EIT) is one such tool, with immense potential for real-time pulmonary function monitoring in neonates. Still relatively new and in the earliest stages of clinical adoption, EIT use in neonatal critical care has been reported in several studies. This review discusses the basic features of EIT, its distinct advantages over traditional pulmonary function monitoring tools, the scope of its adoption in neonatal clinical practice, challenges associated with clinical adoption, and prospects for future applications.

**Impact:**

1. Individualized care assisted by bedside pulmonary function monitoring can positively impact neonatal critical care and outcomes.
2. Electrical impedance tomography (EIT) has the potential to improve neonatal pulmonary function monitoring and treatment outcomes.
3. Electrical impedance tomography can be adopted as a part of routine neonatal respiratory critical care, especially in the population of patients most at risk for bronchopulmonary dysplasia and acute respiratory complications.

## Introduction

In the late 20th century, advancements in understanding newborn lung physiology and pathophysiology, specifically relating to alveolar surface dynamics, along with the development of mechanical ventilation systems highlighted the need for real-time, comprehensive evaluation of neonatal and infant respiratory function. Clinical studies showed that monitoring pulmonary function helped in optimizing lung expansion while avoiding hyperinflation, preventing complications like bronchopulmonary dysplasia, pneumothorax, and intraventricular hemorrhage in the process.^1–3^

## Unique characteristics of the respiratory system in neonates

Neonatal pulmonary function differs significantly from older children. It is characterized by sporadic and cyclical breathing patterns with increased incidence of apnea which reflect an immature respiratory regulatory mechanism.^4,5^ Anatomical differences such as relatively greater dead space and higher compliance of the upper respiratory airway in neonates contribute to increased collapsibility during vigorous inhalation.^6,7^ In preterm newborns, alveoli are fewer and lack collateral ventilation pathways, increasing the risk of overinflation and atelectasis.^8,9^ Furthermore, the neonatal chest wall is more compliant, retracting during inhalation, while the flattened diaphragm and horizontal ribs reduce lung expansion and diaphragmatic efficiency. Both preterm and term infants have a lower proportion of type 1 muscle fibers, contributing to respiratory muscle fatigue and increased vulnerability to distress.^10,11^ Finally, the lack of surfactants in preterm neonates exacerbates the work of breathing and leads to respiratory failure.^12^

Neonatal ventilation is uniquely challenging due to the immaturity of preterm neonatal lungs, which are highly susceptible to ventilator-induced lung injury (VILI). This remains a significant contributor to bronchopulmonary dysplasia (BPD), the most common morbidity in preterm infants. While mechanical ventilation is often essential for sustaining life, it carries inherent risks, including volutrauma, barotrauma, atelectotrauma, and biotrauma, as well as oxidative stress from oxygen toxicity.^13,14^ Advancements in technology have aimed to mitigate these risks through lung-protective strategies that regulate tidal volumes, airway pressures, and positive end-expiratory pressure (PEEP).^14^ However, understanding and addressing the unique physiological mechanics of neonatal lungs and chest walls, including their compliance and interaction as a system, remain critical. Current methods of assessing neonatal lung function such as transpulmonary pressure measurement using esophageal manometry offer valuable insights by separating lung and chest wall mechanics, but these techniques require complex setups and are not compatible with bedside use, particularly in unstable neonates.^15–17^ This limitation highlights the need for more precise, non-invasive technologies to assess lung mechanics in real time. Emerging tools, such as electrical impedance tomography (EIT) hold the promise of providing tailored respiratory support, reducing lung injury, and improving outcomes for preterm infants.

## Electrical impedance tomography (EIT)

The first electrical impedance imaging system for use in humans was described in 1978 by Henderson and Webster.^18^ Subsequently, the case for the use of electrical impedance tomography as a clinical imaging tool was made by Brown et al. in their pivotal paper which detailed properties of EIT that supported its utility in clinical imaging such as high-speed data collection and sensitivity to small changes in physiological phenomena.^19^ Since then, EIT has generated significant interest and has been explored as a tool with vast potential and application as far as diagnostic medical imaging.

### Principles of EIT

Electrical impedance tomography is a non-invasive technique of generating information on organ morphology and function using measurements of the spatial distributions of resistivity (impedance to the flow of electric current) of tissues obtained from electrodes attached around the circumference of the part of the body under study.^19,20^ Inherent variations in the amount of impedance to electric current by different body tissues form the basis of electrical impedance tomography.^19,21^ The resistivities of biological tissues vary over a wide range, from cerebrospinal fluid with a relatively low resistivity of approximately 0.65 ohms-meter to cortical bone with a resistivity greater than100 ohms-meter.^19,22,23^ The amount of substances like fluid and air within organ spaces contribute to biological tissue resistivity. For instance, lung tissue resistivity correlates highly with lung air volume, changing between a resistivity of 7 ohms-meter to 24 ohms-meter between expiration and inspiration respectively.^19,24^ The increase in lung resistivity during inspiration is primarily attributed to the expansion of lung volume with air, which is a poor electrical conductor. This expansion causes the alveoli to elongate and thin, the result being impairment of electrical current conduction through lung tissue.^25,26^ In contrast, increase in the volume of fluid in the lungs (e.g. pulmonary edema) would result in a drop in resistivity.^26,27^ These variations in measured electrical impedance, resulting from inherent differences in tissue properties or pathological processes within the tissue, form the foundation for the application of Electrical Impedance Tomography (EIT) in clinical imaging.

**Fig. 1:**
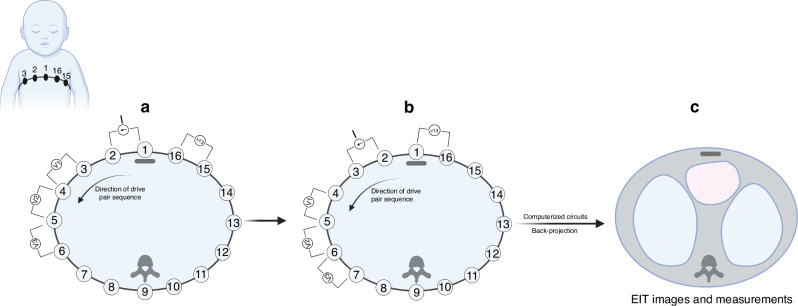
An illustration of the Sheffield stimulation pattern for chest EIT data collection on a cylindrical volume conductor model. Inset – A representation of electrode placement on an infant during EIT chest examination. In the first step (**A**), a low frequency, small voltage, alternating current, I, is injected between electrodes 1 and 2 (the drive pair). Thirteen voltage measurements are simultaneously measured between the remaining electrode pairs 3-4, 4-5... and 15-16. In the second step (**B**), current is injected between the next drive pair in the series, electrodes 2 and 3, while corresponding voltage measurements are obtained from the remaining electrode pairs 4-5, 5-6... and 16-1. This process is continued sequentially until current is injected through all 16 electrode pairs to complete one cycle of measurement. Next (**C**), measured voltages are transmitted through a series of computerized circuits which store collected data, calculate resistivities from measured voltages and convert these through the back-projection algorithm into EIT images and measurements. Created in BioRender. Ako, A. (2025) https://BioRender.com/m88w965. Illustration based on Brown and Seagar^35^ and Malmivuo and Plosney.^29^

To illustrate the basic principles of EIT, we describe the set up for chest EIT examinations. The basic EIT setup has two components – a data acquisition device which is the hardware component and an image reconstruction software.^19,28,29^ The data acquisition system consists of an array of equally spaced electrodes placed around the circumference of the patient’s chest or embedded in a belt wrapped around the chest. Commonly, EIT devices use 16 electrodes, but higher and lower numbers of electrodes have been reported.^30–32^ Increasing the number of electrodes results in a higher image resolution but also lower precision (increased noise-to-signal ratio), increased processing time and increased contact impedance.^28,33,34^ The process of measuring tissue resistance involves transmitting low frequency, small voltage, alternating electric currents in a cyclical pattern between electrode pairs, while the resulting voltages between all other adjacent pairs are recorded.^29,35^ Impedance estimates are calculated from the measured voltages between electrode pairs. For each current stimulation, the electrode pair between which current is injected is called the *drive pair* (Fig. 1). To complete one cycle of voltage measurements, current is injected sequentially through all 16 electrodes pairs in a circular pattern. This pattern of current application and data acquisition is called the *neighboring method* or the *Sheffield stimulation pattern* and is the most widely used, although other methods which may hold technical advantages over it have been described including the *cross*, *opposite* and *adaptive methods*, all described in detail elsewhere.^29,35^ Next, using back-projection algorithms, the collected impedance data is used to reconstruct two-dimensional images (called tomograms) which approximate the spatial distribution of tissue resistivity in the cross section of the body under study.^19,29^

## Core processes involved in chest EIT examination

**Fig. 2:**
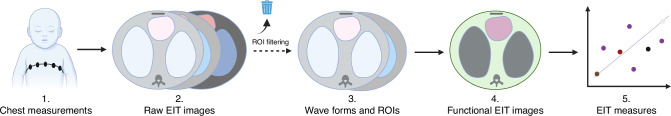
Core processes involved in EIT chest examination. In stage 1, equally spaced electrodes are placed around the chest circumference in the region of interest and small, alternating currents are injected sequentially through electrode pairs. In stage 2, raw EIT images are generated from data frames obtained in the previous step. Stage 3 involves filtering out images which are non-representative of the physiologic phenomena of interest. In stage 4, mathematical operations are applied to the raw EIT images isolated in stage 3 to generate functional EIT images that describe specific physiologic attributes. In stage 5, quantifiable clinically relevant measures that assess physiologic phenomena are generated from functional EIT images. Created in BioRender. Ako, A. (2025) https://BioRender.com/k85n557. Illustration based on Frerichs et al.^36^

The TRanslational EIT developmeNt stuDy group (TREND), an international collaborative working on the clinical promotion of EIT published a consensus paper which proposed a classification system for core processes involved in EIT chest examinations, analysis of data, and a unified nomenclature for EIT parameters.^36^ Five core processes (Fig. 2) are involved in EIT chest examinations and data analysis as follows:

1. Chest measurement: This involves placement of equally spaced electrodes around the circumference of the chest in a position that is most sensitive to the event of interest, usually in the transverse plane (sometimes oblique). Small alternating currents are applied sequentially through pairs of neighboring electrodes (called *drive pair*) while the resulting voltages are measured between the remaining electrode pairs (called *voltage pairs*).^36^
2. Raw EIT images: This consists of generating EIT images from data obtained over one cycle of current application (called *frame*). The number of frames obtained, and thus raw EIT images generated per second is the EIT scan rate.^36^
3. Waveforms and regions-of-interest (ROI): The process of digital frequency filtering of EIT waveforms (generated from raw EIT images) to isolate those representing changes in electrical impedance due to physiological or pathological process of interest from other simultaneously occurring events. For instance, EIT lung region of interest (ROI) can represent regions of ventilation-related impedance changes, separated from changes resulting from cardiac activity or pulmonary circulation.^36^
4. Functional EIT images: The process of applying mathematical operations to a series of raw EIT images and their corresponding pixel waveforms to calculate values that are then plotted into EIT images that describe specific physiological attributes like volume differences and silent spaces.^36^
5. EIT measures – This involves generating quantifiable clinically relevant measures based on functional EIT images to characterize physiologic phenomena like tidal volumes, spatial distribution of ventilation, and regional compliance.^36,37^

Understanding EIT processes and discourse in general requires a familiarity with basic EIT nomenclature and technical terms. Table 1 outlines common terms associated with EIT which are referenced in this paper or may aid understanding of concepts discussed within it. A detailed list of EIT terminology is provided in the supplementary material of the consensus paper on chest EIT examination by the TRanslational EIT developmeNt stuDy group (TREND).^36^

**Table 1:** Common terms and definitions relating to electrical impedance tomography^a^.

Absolute resistivity	An absolute EIT measure that is obtained by determining the difference in resistivity measurements from a human subject to those obtained from a personalized three-dimensional human thorax model.^119,120^ Similar to absolute EIT, absolute resistivity is an experimental concept that has not been widely reported in studies.
Arbitrary units (AU)	A unit of measurement commonly used in EIT to quantify the amplitude of impedance change within EIT image *pixels*.^36^
Contact impedance (electrode skin contact impedance)	The impedance to electric current flow across *EIT device* electrodes and the body. Low contact impedance is necessary to obtain accurate EIT raw data.^36^
Dynamic image	Series of image slices displayed in a movie-like style which show continuously updated relative impedance changes within the *electrode plane*.^36^
EIT device	The electronic system which applies current stimulation through the electrodes attached to the body and records the resulting EIT data.^36^
EIT raw data (EIT data set)	A record of voltages obtained during EIT measurements and the corresponding measurement settings. The set of raw data obtained during one measurement cycle of current application is called a *frame*.^36^
Electrode plane	The plane which intersects the centers of the EIT electrodes attached to the body.^36^
Frame	The set of raw data obtained over one cycle of electric current application. One data frame contains the information needed to reconstruct one *raw EIT image*.^36^
Frame rate (or scan rate)	Number of frames obtained per second. Most EIT devices are designed to allow a maximum scan rate of 50 images per second or 50 Hz.^36^
Functional EIT image	EIT image which describes a specific physiological attribute. It is generated by applying mathematical operations to a series of *raw EIT images* and their corresponding pixel waveforms.^36^
Global information	An EIT measure calculated from an EIT image which is representative of the entire EIT *electrode plane*.^36^
Impedance waveform (or impedance signal)	Impedance change plotted as a function of time. Can be a global, regional or pixel waveform.^36^
Pixel	The smallest image element in a 2-dimensional EIT image. The smallest image element in a 3-dimensional EIT image is called a voxel.^36^
Raw EIT image	The EIT image generated from one *frame* (raw data obtained over one cycle of current application).^36^
Reference frame (baseline)	The reference measurement against which impedance change is measured in dynamic EIT imaging such as time-difference and frequency-difference EIT. The baseline can be defined as a point-in-time measurement such as at end-inspiration or end-expiration, mean EIT data or a moving average of EIT data.^36^
Region of interest (ROI)	A selected subset of an EIT image. The EIT *waveform* in the ROI is the sum or average of the pixel waveforms for all *pixels* in the ROI.^36^
Regional information	An EIT measure calculated from a ROI within an EIT image.^36^
Stimulation current	The electrical current applied to the body during EIT scanning, usually around 1–10 mA and 1 kHz-1 MHz frequency.^36,121^
Stimulation pattern (drive pattern)	The order in which *stimulation current* is applied to the EIT electrodes attached to the body. The most common pattern in use is the neighboring method or adjacent current stimulation pattern where current is applied in sequence through adjacent pairs of electrodes.^29,36^
Tidal variation image, *also*, tidal ventilation map	A functional EIT image that represents the regional distribution of tidal volume. Each *pixel* of this image represents the difference in impedance between end-inspiration and end-expiration.^36,122^

Adapted from Frerichs et al.^36^

^a^Terms defined elsewhere in table are represented in *italics*.

## Techniques for data sampling and analysis

Three data sampling and analysis techniques for EIT image reconstructions have been described: time-difference EIT, absolute EIT and frequency-difference EIT.

*Functional EIT* (f-EIT), also called *time-difference EIT*, is an image reconstruction technique which calculates images of the change in tissue properties between a baseline measurement (reference frame) and a current frame.^36^ It is based on relative resistivity between time intervals, thus generating a dynamic EIT image that shows temporal changes in resistivity distribution. Time-difference EIT is suitable for assessing time-varying phenomena like volume changes during ventilation and perfusion (for example regional and global tidal volumes, end-expiratory lung volumes, lung compliance, pulmonary perfusion), gastric secretion and emptying or the detection of neural activity.^36^ This technique has limited utility in the study of lesions whose compositions do not change over a short period of time (e.g. solid tumors).^38^

*Frequency-difference EIT* (fd-EIT), also called *multifrequency EIT* or EIT spectroscopy, involves injecting alternating currents over a broad band of frequencies and then reconstructing an image which represents the difference in the properties of tissue between two stimulation frequencies. This technique relies on calculating frequency-dependent differences in the characteristics of tissues at a given time.^21,38,39^ Unlike f-EIT which provides a measure of relative resistivity, fd-EIT provides a measure of absolute resistivity and has been used in the study of lung tissue resistivity in neonatal lungs^40,41^ and lung resistivity changes related to pulmonary perfusion.^42,43^

*Absolute EIT* calculates resistivity distribution on an absolute scale. Hahn et al. ^44^ demonstrated a method of obtaining absolute resistivity distribution of artificially induced pneumothorax and hydrothorax in pig animal models using f-EIT generated data and a modified simultaneous iterative reconstruction technique (SIRT).^44^ Similarly, the absolute resistivity of lung tissue was previously estimated by Brown et al. using fd-EIT generated data and a 3-dimensional finite difference model of the thorax as a reference.^39^ These techniques, although more complex than f-EIT and less frequently used, demonstrate methods of deriving estimates of absolute resistivity of body tissue and be applied in a wide variety of clinical studies including of lung volumes and respiratory complications like pneumothorax and solid tissue properties, among others.^39,41,44^

Absolute and frequency-difference EIT generate static images which provide one-time snapshots of absolute resistivity distribution. Compared to dynamic imaging, static imaging is subject to relatively larger measurement errors due to noise and artefacts and exhibit a higher sensitivity to electrode contact quality which necessitates strict requirements for data quality, electrode positioning and EIT technology.^36,39^ While not as commonly adopted in practice as dynamic imaging for the reasons discussed above, static EIT systems have shown potential in detection of soft tissue lesions.

## Key electrical impedance tomography measures and their clinical applications

To effectively understand EIT and its role in neonatal care, clinicians must understand key EIT parameters and how to interpret them in a clinical context. Several EIT measures have been investigated in pre-clinical and clinical studies. In an international survey of clinicians with experience in EIT applications in neonatology and pediatrics, Frerichs and Becher identified some EIT measures which the survey respondents rated as highly useful in clinical neonatology.^37^ These EIT measures included the global inhomogeneity index, ventro-dorsal center of ventilation, silent spaces (low tidal variation regions) and change in end-expiratory lung impedance; measures which mainly assess the homogeneity of ventilation distribution. In critically ill, mechanically ventilated neonates, achieving adequate and safe levels of ventilation as well as more homogenous distribution of ventilation is crucial for minimizing ventilator-associated lung injury.^45^ Measures like tidal impedance variation and regional compliance were considered clinically useful but less so than measures of ventilation distribution with questions arising about reliability of measurements or the availability of alternative traditional approaches such as tidal volume estimation using conventional ventilators. Table 2 outlines some key EIT measures, descriptions and notes on clinical correlations and applications.

**Table 2:** Key electrical impedance tomography measures and notes on clinical applications.

*EIT measure*	*Description*	*Clinical application*
Center of ventilation (CoV)	A measure of the deviation of ventilation distribution from a geometric center relative to the ventral-to-dorsal or right-to-left chest diameter.^36^	Usually expressed as a percentage, a value of 50% represents equal distribution of ventilation between the dorsal and ventral regions while a value less than 50% represents ventilation distribution shift towards the dorsal lung.^36,123^
End-expiratory lung impedance change (ΔEELI)	The difference in end-expiratory lung impedance between two time points. This value can be global or regional.^36^	It correlates with end-expiratory lung volume change. Regional alveolar recruitment can be described as an increase in regional end-expiratory impedance change.^124,125^
End-expiratory lung impedance (EELI), also called, end-expiratory level	A measure of absolute resistivity distribution at the end of expiration, which theoretically correlates with end-expiratory lung volume. A static EIT measure, the commonly used time-difference EIT imaging mode does not calculate this value directly.	Studies have attempted to extrapolate its value from relative EIT measures like tidal impedance variation and corresponding changes in tidal volume.^126^ More commonly, *end-expiratory lung impedance change (ΔEELI)* is used to study end-expiratory lung volume change.^124,126^
Global inhomogeneity index (GI)	This is a measure of tidal volume distribution within the lung. To estimate GI, first the median value of the impedance change represented by each pixel in a tidal variation image is calculated. The sum of the absolute difference between this median value and impedance values of each pixel in the image is then calculated and divided by the sum of the impedance values of each pixel to obtain a normalized estimate. Higher percentage numbers indicate greater inhomogeneity of lung aeration.^123,127^	A normalized estimate, GI exhibits good reliability and interindividual comparability.^122,127^ The higher the GI, the less homogenous tidal volume distribution is. A normal GI value would be around 0.45 - 0.55. A GI of zero would indicate a perfectly homogenous tidal volume distribution while higher than normal values would indicate significant inhomogeneity of ventilation.^127^ GI has been shown to have high correlation with the degree of regional lung recruitment.^101^
Intratidal gas distribution (ITV)	This measure describes changes in regional compliance during a single breath in different regions of interest. Impedance changes in a region of interest from the beginning of inspiration up to a certain time point is expressed as a fraction of the global impedance change.^128,129^	Intratidal gas distribution monitoring can be used to assess recruitability and may guide optimal positive end-inspiratory pressure titration.^129^
Regional respiratory system compliance (C_r_)	An EIT measure calculated by dividing regional tidal impedance variation and driving pressure (airway pressure above PEEP), both monitored simultaneously. Pixel pressure-volume curves can be generated from pixel waveforms and airway pressure. Differences in *compliance* by lung region indicate inhomogeneity of ventilation.^128,130^	During EIT, derecruitment is indicated by decrease of regional compliance and vice versa.^130,131^
Regional ventilation delay (RVD)	Measured during a slow inflation maneuver, it is obtained by dividing the time from global start of inspiration to reaching 40% of the maximal regional impedance change by the total inflation time.^128,132^	Measures temporal delay in regional ventilation which exists in atelectatic areas. It has shown significant correlation with degree of alveolar recruitment.^132^
Regional ventilation delay inhomogeneity (RVDI)	This is the standard deviation of RVD in all pixels. The smaller the RDVI, the more homogenous the distribution of ventilation.^133^	RVDI is a measure of the temporal heterogeneity of ventilation. Like RVD, it correlates well with degree of alveolar recruitment and has been suggested as a useful estimate of cyclic tidal recruitment and collapse that should be minimized to prevent ventilation-induced lung injury.^133^
Silent spaces	These are areas of low tidal impedance variation, defined as areas with impedance change <10% of the maximum.^134^	Silent spaces indicate poorly ventilated lung regions which could represent areas of atelectasis or overinflation.^134^
Tidal impedance variation (TIV), *also* global tidal impedance variation (Global TIV)	The difference between the maximum and minimum impedance values at end-inspiration and end-expiration. Can be regional or global. Global TIV is the sum of impedance changes in all *pixels* in the tidal variation image (see Table 1).^36,128^	TIV represents impedance change (increase) due to inspired air during a tidal breath. Global TIV correlates with tidal volume.^135^ Has been used in the study of effect of repeated surfactant dosing on ventilation.^56^
Ventilation ratio	A functional EIT measure used to quantify the distribution of ventilation in the anterior-posterior (ventro-dorsal) or right-to-left direction. It is calculated as the ratio of the sum of ventilation image pixels in one half (anterior half, right lung, or right half of one lung) of a tidal variation image to the corresponding value in the opposite half (posterior half, left lung, or left half of same lung) of the same tidal variation image.^36^ Sometimes, “fractional ventilation” is calculated instead and is ratio of one half of the tidal variation image to the whole image.^41^	A ratio close to 1 indicates relatively even ventilation, a ratio significantly less than 1 suggests left lung predominant ventilation and vice versa.^136^ This measure may be applied to diagnosing and studying the same clinical conditions as CoV, pneumothorax,^136^ ventilation-perfusion mismatch.^108,137^

## Clinical applications of electrical impedance tomography in neonatology

Although the core concept of electrical impedance tomography (EIT) has existed for decades, its adoption in clinical practice remains in the early stages, with most applications currently limited to clinical research. However, the growing need for bedside monitoring of lung function to individualize patient care, coupled with the increasing commercial availability of EIT devices, has spurred a rise in EIT research and a gradual integration into routine clinical use.^36,37^ Electrical impedance tomography offers several unique advantages, including its compatibility with bedside use, posing minimal risk, providing high temporal resolution, being sensitive to relatively small physiological changes and being non-radiating. These features position EIT as a promising tool to address common challenges in neonatal intensive care units (NICUs), such as diagnostic errors, delays in treatment, and the excessive radiation exposure associated with traditional imaging techniques like X-rays and computed tomography (CT).^46–49^ Newborns are especially sensitive to radiation due to their small size and high mitotic activity, underscoring the need for safer imaging alternatives.^50^

In addition to its imaging capabilities, EIT provides quantitative measures that are especially useful in neonatal care. The cyclical changes in electrical impedance within lung parenchyma during each breath make the pulmonary system particularly well-suited for time-difference EIT examinations. Studies and case reports have explored various clinical applications of EIT in neonatology, covering areas such as: feasibility and safety, which assess the suitability and potential risks of EIT use in neonatal populations; study of lung volume changes in response to therapeutic interventions and supportive care practices; and the use of EIT to guide positive end-expiratory pressure (PEEP) titration in preparation for transitioning from invasive to non-invasive ventilation. Additionally, EIT has been shown to have diagnostic capabilities for frequently seen pulmonary system complications like pneumothorax, atelectasis, and malpositioned endotracheal tubes. These studies highlight the broad potential of EIT to improve both diagnostic accuracy and therapeutic outcomes in neonatal intensive care units.

### Feasibility and safety studies

In a pioneering study, Frerichs et al. studied the applicability and clinical utility of EIT in monitoring regional lung function in pediatric patients aged between 1 day to 7 years who were spontaneously breathing or mechanically ventilated.^51^ Their study was able to identify changes in regional lung volumes resulting from PEEP and other ventilator settings adjustments, surfactant treatment and position change, demonstrating the potential of EIT for clinical application in this patient population.^51^ Riedel et al. successfully used EIT to assess ventilation distribution differences in preterm and term-born infants which were otherwise not detectable by multiple breath washout technique (a technique of lung function testing that measure how efficiently the lungs clear an inert tracer gas).^52^ Sophocleous et al. evaluated the performance of a 32-electrode textile interface for neonatal EIT (electrical impedance tomography) in preterm infants with an average gestational age of 30 weeks.^31^ Over 72 h, electrode-skin contact impedance was monitored in 30 infants. In 25 infants, impedance changes during tidal ventilation were under 5 ohms, and no electrodes failed within the first 20 min after attachment. This meets the threshold of less than 10-ohm fluctuations required for reliable EIT image reconstruction.^53^ The interface caused no significant discomfort, with only minor redness observed in 6 infants. The study concluded that neonatal EIT measurements could be obtained reliably and safely using the textile electrode belt. Becher et al. demonstrated that long-term EIT monitoring is feasible and safe in 200 infants (25 weeks to 36 months post-menstrual age) at risk of respiratory failure.^54^ Using a textile 32-electrode belt, data collection lasted up to 72 h, with sufficient impedance for image reconstruction 77–98% of the time. Minor skin redness occurred in 20 patients but resolved with adjustments, confirming EIT as a safe tool for long-term continuous lung monitoring.

### Monitoring of lung volume changes in response to surfactant administration

Multiple interventions in critically ill infants on assisted ventilation in the NICU are geared towards improving ventilation homogeneity in underdeveloped and delicate lungs. Chatziioannidis et al. studied lung volume changes in response to surfactant treatment administered within 12 h of life to infants of mean gestational age of 30 weeks with respiratory distress syndrome (RDS) on mechanical ventilation, and observed increased ventilation in the dorsal lung and more homogenous right-to-left ventilation distribution post surfactant treatment, measured by analyzing EIT measures center of ventilation (CoV) and fractional ventilation (a corollary of ventilation ratio) respectively (Table 2).^41^ Kallio et al. studied the impact of repeated surfactant treatments on lung volumes in infants (average gestational age 33 weeks) on invasive ventilation.^55^ Despite improved oxygenation and increased expiratory tidal volume after additional surfactant at 33.5 h of life, EIT showed no significant changes in ventilation distribution, end-expiratory lung impedance, or tidal impedance variation. In a randomized controlled trial, Gaertner et al. found no difference in end-expiratory lung impedance change in infants (26–32 weeks gestational age) randomized to CPAP alone or CPAP with prophylactic surfactant.^56^ Other studies have successfully used EIT to assess effects of surfactant treatment on lung volumes in preterm infants.^57–59^ These studies demonstrate the potential of chest EIT examination in monitoring response to surfactant treatment in critically ill infants.

### Study of lung volume changes with different modes of invasive and non-invasive ventilation

Gaertner et al. used EIT to study regional ventilation in preterm infants (26–34 weeks postmenstrual age) receiving nHFOV or nCPAP in a crossover trial.^60^ They found comparable global tidal volumes (slightly lower during nHFOV) and preferential ventilation distribution to the right lung and non-dependent regions. Similarly, Thomson et al. analyzed ventilation patterns in preterm infants (<30 weeks gestation) on CPAP or HFNC. Both studies noted right lung and non-dependent region preference, with Thomson et al. linking ventilation inhomogeneity to increased risk of BPD.^61^ Virsilas et al. compared lung volumes in preterm neonates on invasive ventilation versus CPAP or HFNC, finding higher end-expiratory lung impedance with invasive ventilation but greater ventilation to dependent lung areas with non-invasive methods.^62^ Miedema et al. used EIT to monitor regional lung volume changes in preterm infants on nCPAP and BiPAP, observing a homogenous increase in end-expiratory lung volume (EELV) and tidal volume with nCPAP, while BiPAP increased EELV without affecting tidal volume.^63^ Thomann et al. demonstrated EIT’s ability to detect small oscillatory volumes in infants receiving high-frequency oscillations and nasal high-flow therapy.^64^ Studies by Gaertner and Bhatia have investigated lung volume changes during apnea while on nHFOV or nCPAP, and during extubation from synchronized positive pressure ventilation to nCPAP respectively.^65,66^

### Study of lung volume changes in response to supportive care practices like endotracheal tube suctioning and body position change

Other studies have investigated changes in lung volumes in response to supportive care practices. Studies examining the effects of endotracheal tube suctioning using chest EIT examinations to assess global and regional tidal impedance variations and end-expiratory level change have reported transient acute loss of volume followed by spontaneous recovery of global lung volume with possible residual regional volume loss suggestive of atelectasis. These findings could guide ETT suction practices to achieve safe and beneficial results in high-risk infants.^67–69^ Similarly, EIT measurements of tidal impedance variation (TIV), end-expiratory lung impedance change (ΔEELI), and global inhomogeneity index (GI) have been used to investigate the effects of body positioning and body position change on regional ventilation.^70–74^ These studies show that prone positioning enhances dorsal lung aeration, which can reduce atelectasis, while supine positioning promotes more symmetrical ventilation. Misalignment of the head and body can lead to asymmetries in ventilation, emphasizing the importance of precise positioning during respiratory care. These findings are especially relevant for tailoring supportive care practices in neonates at risk of respiratory complications.

### Positive end-expiratory pressure (PEEP) titration to minimize ventilation inhomogeneity

Zhao et al. demonstrated the feasibility of using EIT measurements of global inhomogeneity index and analysis of intranidal compliance-volume curves to titrate PEEP to optimal levels that allow maximum ventilation homogeneity.^75^ To test the capability of EIT to evaluate ventilation distribution during spontaneous assisted ventilation in neonates on conventional mechanical ventilation and predict successful extubation to CPAP, Rossi et al. performed chest EIT examinations during stepwise, decremental PEEP titration in intubated very low birth weight infants prior to planned extubation.^76^ They successfully analyzed functional EIT images and calculated ventro-dorsal ventilation ratio to determine PEEP levels that resulted in the best ventilation homogeneity just prior to extubation, and in the process demonstrating the potential of EIT to aid and predict successful extubation in critically ill neonates. LaVita et al. in their prospective case series demonstrated the use of chest EIT examinations and measurement of regional ventilation distribution to guide PEEP titration to minimize overdistention and atelectasis in infants of age <6 months with bronchopulmonary dysplasia.^77^

### Diagnosis of pulmonary diseases encountered in neonatology

Research studies and case reports have demonstrated the role of EIT functional images and EIT-measures obtained in real-time in the diagnosis of conditions like lobar atelectasis (decreased tidal impedance variation and loss of impedance amplitude on affected side, increased ventilation inhomogeneity),^78^ pneumothorax (increased end-expiratory lung impedance, decreased tidal impedance variation, decreased end-expiratory lung impedance on contralateral side due to mediastinal shift and lung compression),^79–81^ and congenital hyperinflation of lung lobe (increased right-left and regional ventilation inhomogeneity).^82^

## Challenges associated with EIT use in clinical practice

As mentioned earlier, there are not widely accepted consensus, standardized approaches to the execution of EIT measurements, selection of EIT data and EIT measures for clinical or research purposes. Furthermore, propriety and older algorithms pose a challenge to accurate EIT image reconstruction.^83^ For more routine clinical adoption, standardized protocols to guide clinical applications of EIT still need to be developed and adopted.^36,83,84^ Several other EIT systems are commercially available in some parts of the world.^85–90^ Different image reconstruction algorithms have been developed for use with EIT devices and include the Sheffield-back projection algorithm,^20^ Newton-Raphson reconstruction algorithm,^91^ Particulate Swarm Optimization (PSO),^92^ Graz consensus reconstruction algorithm for EIT (GREIT),^83^ among others. Some classification algorithms to standardize EIT data selection have been proposed but none has yet been universally adopted.^83,84,93^

Measurement errors and artefacts present another challenge to routine EIT use in clinical practice. The measured set of voltages on the surface of the body which forms the basis of EIT data collection is a function of the position of the electrodes, resistivity distribution and shape of the body part under study.^32^ Loss of contact between electrode and skin or loosely fitting electrodes caused by patient movement or change in body position would cause errors in data collection.^94^ Owing to individual differences in the shape of the cross section of the chest cavity, errors may ensue from extrapolating EIT measurements to standard chest shape models as a reference during image reconstruction like in the Sheffield-back projection algorithm.^32^ Thus, EIT chest examination techniques and image reconstruction algorithms that can be individualized to the patient and better approximate lung volumes are being developed.^32^

Compared to lungs and other tissues within the thorax, air generates the highest resistance to current flow, as such, air volume changes associated with tidal breathing accounts primarily for the impedance changes recorded on EIT chest examinations.^25,26,95^ However, in very small premature infants and infants on bubble continuous positive airway pressure (CPAP) ventilation, the contribution to impedance changes due to cardiac activity, including tidal variations in heart rate and filling volume, changes in great blood vessel volume, volume of pulmonary circulation and pressure changes caused by bubble CPAP is significant enough in comparison to impedance generated by tidal breathing.^95^ Manually parsing artefact-free measurements from EIT data is an error prone process.^96^ To prevent errors in interpretation of EIT data and image reconstruction, standardized protocols to guide the filtering of data to parse out artefactual data are needed.^95,97,98^

The spatial resolution of EIT is poor compared to computed tomography (32 ×32 pixels versus 512 ×512 pixels).^99^ However, EIT has a much higher temporal resolution than computed tomography (typically 20–50 Hz vs ≈ 0.3–1 Hz).^99^ This gives EIT a distinct advantage as a bedside monitoring tool that can detect physiologic and anatomic changes of clinical importance in real time.^21,99^

## Prospects for EIT use in neonatology

### Study of neonatal lung physiology during early transition to extrauterine life

Multiple studies using EIT have demonstrated the vital role it can play in improving our understanding of respiratory physiology of newborns. Tingay et al. used EIT to study intrathoracic processes occurring during transition to extrauterine life at birth.^100^ They demonstrated ventilation inhomogeneity immediately after birth as represented by tidal ventilation favoring the right and non-dependent lung regions and demonstrated the role of the initial cry in intrathoracic volume redistribution and preservation of functional residual capacity. Similarly, Gaertner et al. studied intrapulmonary gas flows during different breathing patterns registered in the transition period in preterm infants, identifying in the process the likely role of breath-holding pattern of breathing in increasing end-expiratory lung volume and intrathoracic air redistribution via pendelluft gas flows.^101^ Janulionis et al. compared differences in regional lung ventilation of late preterm and term neonates during transition to extrauterine life using EIT and found more silent spaces in non-dependent lung areas in late preterm neonates during measurements taken within 30 minutes of life.^102^ These studies showcase the role EIT can play in vastly increasing our understanding of neonatal respiratory physiology, neonatal adaptation to extrauterine life and improving delivery room care.

### Study of lung growth and development in the postnatal period

Brown et al. studied trends in absolute resistivity of lung tissue in preterm infants and young children.^103^ They described changes in lung resistivity with age that may reflect maturational changes in number and size of alveoli and lung tissue perfusion, indicating the potential role of EIT in long lung development in the postnatal period.

### Individualization of care in invasive mechanically ventilation and non-invasive respiratory support

Electrical impedance tomography when used to monitor ventilation distribution can guide adjustment of ventilation strategies to optimize pulmonary function and minimize associated injury. A functional tool which allows changes in pathology to be monitored in real time, more routine use of EIT at the bedside can significantly improve care delivery.^82^ During invasive mechanical ventilation, EIT can be primarily useful in guiding the titration of positive end-expiratory pressure (PEEP) and monitoring of lung recruitment, which is crucial for optimizing ventilation while balancing recruitment and overdistension.^25,104^ Electrical impedance tomography may provide a more reliable alternative to conventional tidal volume measurements for patients invasively ventilated using uncuffed endotracheal tubes.^37^ During non-invasive respiratory support, EIT can primarily be used to monitor changes in lung aeration and regional ventilation distribution. It can aid in assessing the effectiveness of CPAP therapy and other non-invasive modalities by providing real-time feedback on ventilation patterns.^104^

### Clinical applications beyond pulmonary function monitoring

The use of EIT in monitoring intracranial hemorrhage was first suggested by Brown et al.^19^. Using electrodes wrapped around the occipitofrontal circumference of a 28-week gestation infant, EIT was able to detect intraparenchymal and intraventricular hemorrhage.^105^ The potential of absolute and multifrequency EIT imaging techniques in tissue differentiation has been reported.^38^ Studies in adults have suggested EIT could be used in the evaluation and diagnoses of epilepsy, stroke, cerebral edema, cerebral abscess and other brain diseases.^106^ The potential for EIT use in monitoring gastric emptying has also been described.^107^

### Monitoring of pulmonary perfusion

Electrical impedance tomography has demonstrated its ability to selectively measure lung perfusion by isolating cardiac-domain signals, independent of heart movement -related and ventilation-related signals, providing insights into regional pulmonary blood flow.^108^ This emerging application highlights EIT’s potential in optimizing care for neonatal conditions associated with ventilation-perfusion mismatching, such as respiratory distress syndrome and pulmonary hypertension.^107,109^

### Pulmonary arterial pressure monitoring

Measurement of pulmonary artery pressure relies on right heart catheterization. Electrical impedance tomography could provide an alternative, non-invasive method to the estimation of pulmonary artery pressures. Studies in adults have shown a correlation between impedance variation of lung perfusion and hemodynamic parameters like stroke volume as well as disease severity and prognosis.^110,111^

### Potential role for artificial intelligence

Artificial intelligence could play a role in EIT data analysis and interpretation. This rapidly developing approach to data analysis could potentially help overcome current challenges with errors of measurement, dealing with artefactual data, improving quality of image reconstruction.^112–115^ Deep learning has significantly enhanced EIT image quality and resolution. However, advancing AI integration requires large, high-quality datasets, which are resource-intensive to collect and process.^113^ Despite these challenges, this rapidly evolving field holds great promises for innovative solutions.

## Electrical impedance tomography in neonatal animal studies

It is important to mention the role studies in neonatal animal models have in the understanding and clinical applications of EIT. Tingay et al. demonstrated how EIT-guided sustained first inflation (SI) tailored to achieve lung volume equilibrium significantly improved gas exchange, enhanced lung mechanics, and minimized lung injury compared to fixed-duration or no SI approaches in preterm lambs.^116^ In another study, Miedema et al. identified predictive changes in ventilation distribution and phase angle delays preceding spontaneous pneumothorax using EIT examinations in preterm lambs, highlighting its potential for early detection and prevention of critical events.^117^ Hochhausen et.al. demonstrated in a study of pigs that EIT examinations can facilitate PEEP titration in ARDS to minimize ventilator -induced lung injury.^118^ Animal studies like these and many others, not only validate EIT as an important research tool but also herald its potential to transform respiratory care in the NICU, guiding individualized and evidence-based approaches to improve outcomes for preterm infants.

## Conclusion

Electrical impedance tomography is a non-invasive, radiation-free imaging technology capable of continuous, bedside monitoring of pulmonary and other physiological processes. It offers significant potential to support clinical decision-making in neonatal critical care, though its routine bedside use remains in the early stages. Adoption is limited globally due to limited awareness among neonatologists, as well as technical challenges such as standardization of EIT protocols, defining EIT metrics, and managing artefactual data. To unlock EIT’s full potential, knowledge sharing and education within the neonatal care community are essential. The integration of artificial intelligence may further accelerate EIT development, enhancing its clinical utility. Additionally, increased accessibility to EIT devices through manufacturers and regulatory support will drive further clinical research, providing deeper insights into its applicability and benefits in neonatal care.

## Data Availability

The production of this review article did not involve any primary data collection, and therefore, no new data were generated or analyzed as part of this study. The article is based solely on a comprehensive literature review of existing publications, which are cited within the text.
